# Changing the Way We Report, Interpret, and Discuss Our Results to Rebuild Trust in Our Research

**DOI:** 10.1523/ENEURO.0259-19.2019

**Published:** 2019-08-01

**Authors:** Christophe Bernard

**Affiliations:** Editor-in-Chief

## Abstract

*eNeuro* is moving forward with a new initiative asking authors to present their results with estimation statistics and not to rely solely on *p* values. In this editorial, I would like to introduce to you the concept of this new statistics while first discussing my evaluation of the present situation and my own experience with using statistics to interpret results, then I will propose a solution and how we will move forward in the journal. I have also included my own experience using these new statistics and provided a list of resources. This new initiative will not change what is already acceptable for statistics in the journal; it is to encourage a simple addition of using estimation statistics.

I am pleased to announce that *eNeuro* is taking a major initiative to improve the way we communicate our results to ourselves and society. This is not a revolution, just applying common sense using a straightforward solution: the estimation approach. *eNeuro* has already required results tables that include either a confidence interval (CI) or observed power. Encouraging authors towards estimation is just an extension/refinement of existing strong practices at the journal. This editorial discusses why we ended up proposing this solution to our community. It is linked to a Commentary published simultaneously (Calin-Jageman and Cumming, 2019), which describes the benefits of the estimation approach. When I was thinking to propose this change in the journal policy, I had a paper under revision at *eNeuro* (Manouze et al., 2019). I seized the opportunity to change the way I was reporting the results, using the estimation approach. I can thus tell you precisely what it takes and what it can bring. So, bear with me, because I would first like to provide my evaluation of the present situation. Otherwise, skip to the proposed solution.

Experimental research is based on observations and measurements. Data interpretation comes next, as we attempt to make sense of these observations and reach some conclusions. The validity of the interpretations and conclusions primarily depends upon whether or not the collected data reflect the reality. The difficulty we face is variability, which can be intrinsic (a unique set of parameters may characterize each living organism and measuring the same parameter across organisms will provide different values) and extrinsic (variability in the experimental or measurement conditions). Statistics were first developed to estimate the basic properties of a country, such as its population. With the development of probability theory, experimentalists had tools to assess how confident they could be regarding their observations, interpretations, and conclusions. Although *eNeuro* is neutral regarding schools of thoughts, e.g. frequentists versus Bayesians, I only wish to comment on frequentist statistics, which is used in the vast majority of neuroscience papers.

When I started my PhD, I received basic training in statistics. However, in my day-to-day experimental life, I was rarely using them. I was collecting data points, displaying all of them, thinking about them, and trying to make sense of them. Statistics, if any, was coming at the end. My first papers did not use statistics, and the reviewers never requested them. Sure, that was the last century, but I do not think that time matters here. The statistical tools of today already existed. What changed is our approach to experimental research.

When I reflect on my scientific trajectory, I realize that I became more and more *p* focused, and anything with a *p* < 0.05 became the truth, not only for my research but also when evaluating that of my colleagues. I do not know when and how the change occurred. Some high-profile journals did play a role when demanding to have significant results and imposing complex statistical reports. Whatever the causes, the result is today’s over-reliance on the *p* value. It shaped our experimental approach and twisted our minds. Many papers have been written on the crisis science is going through, including the loss of faith in scientists from the general public, the decision makers, but also from other scientists. Like me, you must be amazed when reading papers from high-profile journals (but not only from these journals) reporting smooth stories, in which everything works, everything is claimed to be significant, and thus true, with a minimal number of experiments. At best, I am skeptical. The conclusions are very strong, leaving no room for doubt (otherwise you cannot publish in these journals). This practice has contributed in no small way to today’s reproducibility crisis. Here I am not talking about scientists faking results or selecting the results that support a theory; this has always existed. The problem is more profound: we transformed a number that is linked to a probability into a goal to achieve, a definitive answer. In short, we distorted the *p* value to make it say what it is not designed to say. This happened to the journal impact factor, for which the original use has been completely distorted. We all know that the impact factor is not a good metric (for its present use). We did the same thing with the *p* value. It is time to go back to its original meaning. The realization for me was when I learned about the “Dance of the *p* Values” (https://www.youtube.com/watch?v=5OL1RqHrZQ8). This short video explains it all. It was an eye-opener. Although I knew, intellectually, that sampling from two distributions may or may not achieve a “significant” result, I had stopped thinking further or questioning the results when I was getting to the “grail”: *p* < 0.05. The video is a concrete example and a humility lesson. When I am teaching, I now begin every course (at any level) with this video to demonstrate how uncertain we can be about the reality we think we are describing.

Thus, it is time for the scientific community to course-correct, as is the nature and obligation of the community to do when we realize we, as scientists, have let the pendulum swing too far in one direction making us myopically view our results.

## The Solution

Revert to the original truth and accept the fact that our measurements imperfectly reflect the reality. One easy way to more accurately report data is to be open about it and use estimation statistics. In essence, it is just reporting how confident, in a quantitative manner, we are about our observations and estimate the potential error. This allows a much more fruitful discussion of the results and a more accurate report. A critical cautionary note though: estimation statistics is not a panacea; it is not the solution. As for the *p* value, it must not be diverted from its original use. We do not want to use it to say something it cannot say. Another important note: it does not apply to all types of measurements. But interestingly, estimation statistics may be the only tool that both frequentists and Bayesians agree upon. Everything is explained with concrete examples in the associated commentary.

## How to Move Forward

I must confess that I had no idea of what estimation statistics were until I attended the symposium on reproducibility during last year’s annual SfN meeting and Dr. Robert Calin-Jageman’s talk. My only motivation as editor-in-chief is to serve the community and, when needed, use *eNeuro* as a vehicle to build a better world for us. After the talk, I started to think about introducing estimation statistics to the journal. When I discussed this possibility with *eNeuro*’s Advisory Board and Reviewing Editors, they mentioned a major problem: most scientists do not know estimation statistics. Since they are all active scientists, neither do Reviewing Editors and reviewers. Thus, this change in reporting cannot be imposed on authors. The switch should be progressive when we all realize how powerful it is. Hence, while strongly encouraged, the use of estimation statistics (where appropriate) will remain optional at *eNeuro*. Yet, this is a simple way to revert to good practice in data analysis, interpretation, and discussion.

But first, I had to convince myself that it is not too time consuming and too difficult. Since our group had a paper in revision at *eNeuro*, I decided to have a go and redid all the analysis and figures myself. I used Dr. Robert Calin-Jageman’s advice (as described in his commentary) and used the https://www.estimationstats.com/ website to paste raw data directly and obtain all values and figures, or https://thenewstatistics.com/itns/esci/ to download Excel files to perform the analyses. The first website is particularly useful as you can copy-paste the results of the analysis into your paper and download the figure in a vector-based form to compose the figure with your favorite software. It was surprisingly fast and easy. The website has an educational part, which explains how to interpret the results. The book by Cumming and Calin-Jageman, *Introduction to the New Statistics: Estimation, Open Science, and Beyond* (Cumming and Calin-Jageman, 2016), is also excellent and simple; I used it at the beginning. But the fascinating part was to see our data, which provided us with another level of interpretation. Importantly, it provided us with a way to evaluate how confident we can be, in particular, calling for replication when necessary. In fact, by just looking at the CIs, we already knew how confident we could be. The *p* value is only another component of the analysis. Finally, displaying all data points allowed us to discover something we were not expecting, bringing another dimension to the story. Then, what happened during the revision? The reviewers appeared happy with the new presentation as they did not raise further comments. I guess that reviewers and reviewing editors will not be disturbed by the addition of estimation statistics; it is intuitive. Because some of you may wonder, the whole process was double-blind (I have no way to know who the reviewers are, and the reviewers did not know from which laboratory the paper was coming from).

If you decide to try estimation statistics, you will see that it is effortless; in particular, if you use it from the beginning when you start to analyze your results. Late last year, *eNeuro* published a paper in which authors used estimations statistics (Ouellette et al., 2018). The paper from my group (Manouze et al., 2019) another *eNeuro* article in press now (Acharya et al., 2019), and hopefully from many others, will follow in the future.

In conclusion, I hope that I have convinced some of you to try it. As I mentioned, the aim is not to give up the *p* value approach, only to give it back the place it should occupy in our analysis arsenal. The goal is to use a powerful way to be more honest about what our results can tell us. Again, the estimation approach applies to certain fields and conditions. It must be used with knowledge of its limitations. We are going to run the experiment at *eNeuro*, that is, encouraging authors to try the estimation approach (and perhaps directly help them too, providing assistance when necessary) and see what happens. We need your feedback. So we invite you to discuss the pros and cons of various approaches as a function of data structures and neuroscience fields on the blog discussion post: http://blog.eneuro.org/. We need your feedback. Finally, there is another dimension to the estimation approach: the way we plan our research, which I leave you to discover in the Commentary (Calin-Jageman and Cumming, 2019) or the book.

Estimation statistics recommended resources:

- The online community and blog for Introduction to the New Statistics: https://thenewstatistics.com/itns/
- Estimation Statistics Beta site to analyze your data with effect sizes: https://www.estimationstats.com
- Cumming G and Calin-Jageman RJ (2017). *Introduction to the New Statistics: Estimation, Open Science, and Beyond.* New York: Routledge.
- Short video about the dance of the *p* values: https://www.youtube.com/watch?v=5OL1RqHrZQ8
